# The unequal impacts of the COVID‐19 pandemic on young adults' mental health. Predictors of vulnerability and resilience using longitudinal birth cohort data in the UK

**DOI:** 10.1002/jad.12400

**Published:** 2024-08-29

**Authors:** Harriet Reed, Ajay Thapar, Lucy Riglin, Stephan Collishaw, Christopher B. Eaton

**Affiliations:** ^1^ Division of Psychological Medicine and Clinical Neurosciences Cardiff University School of Medicine Cardiff UK; ^2^ Wolfson Centre for Young People's Mental Health Cardiff University Cardiff UK

**Keywords:** COVID‐19, longitudinal study, mental health, resilience, vulnerability, young adult

## Abstract

**Introduction:**

Previous studies have demonstrated deteriorations in young adult mental health during the COVID‐19 pandemic, but evidence suggests heterogeneity in the mental health impacts of the pandemic. We sought to identify factors which may predict changes in psychological distress and wellbeing during the COVID‐19 pandemic in UK young adults.

**Methods:**

A total of 2607 young adults from the Millennium Cohort Study were included. Psychological distress and mental wellbeing were measured using the Kessler‐6 and Short Warwick‐Edinburgh Mental Wellbeing Scale, respectively. Assessment occurred at three timepoints between the ages of 17–19: 2018/19 (pre‐COVID Baseline), May 2020 (COVID Wave 1) and September/October 2020 (COVID Wave 2). Latent change score models were used to study change in distress and wellbeing across the study period, as well as the impact of sex, relative family poverty, parental education, preexisting mental health difficulties and perceived social support on these changes.

**Results:**

The latent change score models suggested both distress and wellbeing tended to increase across the study period. Being female and in relative poverty predicted greater increases in distress and/or poorer wellbeing. Higher levels of parental education and greater perceived social support were protective against increased distress and associated with improved wellbeing.

**Conclusions:**

The impact of the COVID‐19 pandemic on UK young adult mental health is complex. We provide further evidence for a distinction between symptoms of poor mental health and wellbeing. Research is urgently needed to assess the long‐term impacts of the COVID‐19 pandemic on the mental health and wellbeing of young people, particularly in more vulnerable groups.

## 1 INTRODUCTION

Young adulthood is a peak period for the onset of common mental health disorders (Solmi et al., 2022) and three quarters of adults with a diagnosable mental health disorder experience the onset of symptoms before the age of 24 (Kessler et al., 2005). Poor mental health during adolescence and young adulthood are associated with physical health problems, psychiatric disorders and poorer social, educational and economic outcomes (Gibb et al., 2010; Johnson et al., 2018; Thapar et al., 2012). The prevalence of mental health problems is also increasing for recent generations of young people. Studies using unselected United Kingdom and international population cohorts have shown a substantial increase in emotional disorders and symptoms in young people, including depression and anxiety, especially in females over recent decades (Collishaw & Sellers, 2020; Sadler et al., 2018; Sigfusdottir et al., 2008). The onset of the COVID‐19 (SARS‐CoV‐2) pandemic resulted in restrictions being introduced designed to suppress the spread of the virus in the United Kingdom on 23rd March 2020, for example the closure of schools, workplaces, and business, as well as social distancing. Evidence suggests this further impacted the mental health of children, adolescents, and young adults (Viner et al., 2022). It is likely that impacts of the pandemic on mental health have varied substantially according to individual circumstances, available support structures, and preexisting vulnerability factors.

Studies focusing on the immediate impacts of the COVID‐19 pandemic in the United Kingdom (March–May 2020, covering the period of the first lockdown), and which have compared change in mental health relative to a pre‐COVID baseline provide evidence for a decline in young adult mental health. Kwong et al. (2021) analyzed responses over time from a variety of mental health screening tools in the Avon Longitudinal Study of Parents and Children (ALSPAC), with the COVID‐19 assessment period taking place between 9th April and 14th May 2020. They found that the percentage of adults (mean age: 28 years) with probable anxiety disorder and poor well‐being almost doubled (12.9%–24.3% and 7.6%–13.3%, respectively), relative to a pre‐COVID‐19 baseline (the median length of time between pre‐COVID and COVID‐19 assessments was 2–7 years). Interestingly, rates of probable depression decreased during the pandemic; from 24.4%–18.1%. The United Kingdom Household Longitudinal Study, UKHLS (Pierce et al., 2020), found that young people aged 18–24 years had a mean score on the General Health Questionnaire (GHQ‐12) that was 2.69 points higher than predicted according to prepandemic trends, indicating poorer mental health. Finally, Wiedemann et al. (2022) examined ~600 young adults aged 19–34 from the Neuroscience in Psychiatry Network cohort study and used the Kessler Psychological Distress Scale (Kessler et al., 2002) and Short Warwick‐Edinburgh Mental Wellbeing Scale (Stewart‐Brown et al., 2009) to model individual trajectories of psychological distress and mental wellbeing, respectively. They observed that 8 in every 10 individuals showed higher psychological distress scores and lower mental wellbeing scores during the first national lockdown (May 2020) than expected, based on previous waves of assessment (2012–2017).

Several studies evaluating mental health beyond the initial period of national lockdown suggest a more complex picture. Daly et al. (2022) observed that the greatest increase in the prevalence of mental health problems between 2017 and 2019 and April 2020 was in 18–34‐year‐olds in the United Kingdom. However, interestingly, this age group also demonstrated the largest reduction in mental health problems when remeasured in May and June of 2020. Further support for these findings come from a study of 18–25‐year‐olds from the UKHLS (Stroud & Gutman, 2021). Growth curve modeling of young adult mental health showed that GHQ‐12 scores were highest in April 2020, then subsequently improved over the spring and summer months, before worsening again from September 2020. Rosa et al. (2022) similarly found that depressive symptoms decreased in young adults from the Millenium Cohort Study between May‐September 2020, but had increased again by the time of February/March 2021, with symptom scores being higher than they were in May 2020. These temporal variations in mental health symptom scores coincide with the easing and tightening of UK lockdown restrictions over this period (Stroud & Gutman, 2021).

Studies conducted outside of the United Kingdom and with age groups also encompassing children and adolescents suggest a decline in mental health during the COVID‐19 pandemic. Studies with young people in the Netherlands, Germany, the United States, Canada, and Israel observed declining mental health during the COVID‐19, pandemic, relative to a prepandemic baseline (Alt et al., 2021; Deng et al., 2021; Hollenstein et al., 2021; Luijten et al., 2021; Romm et al., 2021; Sabato et al., 2021). Other studies based in Canada, China, and Sweden did not observe significant worsening of adolescent mental health secondary to the pandemic (Bélanger et al., 2021; Chen et al., 2021; Johansson et al., 2021; Lu et al., 2021; Vira & Skoog, 2021), whilst other studies noted mixed findings, with some symptoms of poor mental health decreasing from before to during the COVID‐19 pandemic (Bernasco et al., 2021; Hollenstein et al., 2021).

Wolf and Schmitz (2023) and Kauhanen et al. (2023) reviewed studies comparing the mental health and wellbeing of young people before and during the COVID‐19 pandemic (69 studies from 21 countries and 21 studies from 11 countries, respectively; age range across both reviews: 3–24 years). Both reviews point toward an overall decline in mental health and wellbeing for young people during the COVID‐19 pandemic, with heighted psychological stress and increased depression and anxiety symptoms, as well as increased loneliness.

It is also likely that young people differed in their experience of the pandemic due to variation in preexisting vulnerability and resilience factors that might have buffered or exacerbated the impacts of the pandemic on young people's mental health. Studies in the United Kingdom and internationally suggest that young people's mental health and wellbeing across the COVID‐19 pandemic may have been associated with factors such as their gender, the presence of a mental health condition, neurodevelopmental disorder, chronic illness, feelings of loneliness, their capacity for emotional self‐regulation, family socioeconomic status, health‐related worries, consistent routines and structure, parental mental health and level of social support from family and friends (Alt et al., 2021; Bernasco et al., 2021; Campione‐Barr et al., 2021; Deng et al., 2021; Di Giunta et al., 2021; Ellwardt & Präg, 2021; Hollenstein et al., 2021; Jiang et al., 2021; Kwong et al., 2021; Magson et al., 2021; O'Connor et al., 2021; Pierce et al., 2020; Ravens‐Sieberer et al., 2021; Romm et al., 2021; Rosa et al., 2022; Sabato et al., 2021; Schmuck et al., 2021; Shakeshaft et al., 2023; Shi & Wang, 2021; Shoshani & Kor, 2021; Stroud & Gutman, 2021; Wiedemann et al., 2022; Wolf & Schmitz, 2023). Identifying risk and protective factors for young people's mental health during the COVID‐19 pandemic is important for future efforts to prevent poor mental health during similar crises (Kauhanen et al., 2023).

Our overall objective was to explore predictors of mental health and wellbeing outcomes across the COVID‐19 pandemic, within young adults living in the United Kingdom. Specifically, we aimed to:

1. Test changes in psychological distress and wellbeing scores across the COVID‐19 pandemic in young adults from the Millennium Cohort Study (MCS). We include three timepoints: 2018/19 (pre‐COVID Baseline), May 2020 (COVID Wave 1) and September/October 2020 (COVID Wave 2).
2. Identify subgroups of young adults in the United Kingdom who may vulnerable to, or resilient against, poorer distress and wellbeing outcomes during the COVID‐19 pandemic.

## 2 MATERIALS AND METHODS

### 2.1 Sample

The MCS is a multidisciplinary survey study, following the lives of young people born across the United Kingdom between 2000 and 2002 (Connelly & Platt, 2014). The current study focuses on a core sample of 2607 young adults from MCS who took part in the first wave of online surveys during the COVID‐19 pandemic: COVID Wave 1, CW1 (see https://cls.ucl.ac.uk/covid-19-survey/). This wave took place from 4th to 30th May 2020, when participants were on average 19 years old. Young adults and parents who had not withdrawn from the MCS cohort, who were traceable and who were known not to have died were invited to take part in the surveys by email (Brown et al., 2020). For the first COVID‐19 survey, the issued sample was 9946 and the response rate was 26.6% (*N* = 2645).

For participants in our core sample, we also included data from the second COVID‐19 survey (which took place from 9th September to 11th October 2020, COVID Wave 2, CW2) and from a pre‐COVID assessment: “Baseline” (Sweep 7 of the MCS, which took place from 8th January 2018 to 8th April 2019, when participants were on average 17 years old). For participants in our core sample, we also included data on variables which may associate with changes in distress and wellbeing; provided at birth, at Sweep 6 of the MCS (January 2015 to March 2016), when participants were on average 14 years old and during the CW1 and CW2 assessments. It is important to note that when participants were assessed at CW1, this coincided with restrictive “stay at home” measures implemented in the United Kingdom, to control the spread of the COVID‐19 virus, although during this month individuals who were unable to work from home were being advised to start to return to work. At CW2, participants would have still been experiencing restrictions, although generally these were less stringent, including for example limiting indoor and outdoor gatherings to six people and home working.

MCS received ethical approval from the London Multi‐Centre Research Ethics Committee. More information is available at: http://www.cls.ioe.ac.uk/.

### 2.2 Mental health and wellbeing

Psychological distress and mental wellbeing were assessed at Baseline and in CW1 and CW2 using the Kessler‐6 (K6) scale (Kessler et al., 2002) and the Short Warwick‐Edinburgh Mental Wellbeing Scale (SWEMWBS) (Stewart‐Brown et al., 2009). These scales measure psychological distress and mental wellbeing, respectively, and both have been shown to be valid and reliable (Kessler et al., 2003; Umucu et al., 2021; Vaingankar et al., 2017). For example, the K6 was shown to be an unidimensional measure of psychological distress across groups with anxiety disorders, bipolar disorder and schizophrenia and to show concurrent validity with measures of distress impact. The K6 includes 6 items pertaining to symptoms of depression and anxiety experienced in the last 30 days. Total scores for the K6 range from 0 to 24, with higher scores indicating greater psychological distress. The SWEMWBS consists of 7 items that reflect positive wellbeing in the last 2 weeks. Total scores for the SWEMWBS range from 7 to 35, with higher scores indicating greater mental wellbeing.

### 2.3 Vulnerability and resilience factors

We assessed whether the following variables were associated with variation in change in mental health and wellbeing scores over the three waves of assessment (pre‐COVID Baseline, CW1, CW2): sex, relative family poverty, parental education, preexisting mental health and/or behavioral difficulties and perceived social support.

Preexisting mental health and/or behavioral difficulties were assessed at age 14 using the parent‐rated Strengths and Difficulties Questionnaire (SDQ) (Goodman, 2001). This is a well‐validated screening tool for child mental health and behavioral difficulties, assessing emotional symptoms, conduct problems, hyperactivity/inattention, and peer relationship problems. Total scores range from 0 to 40. In line with previous studies exploring youth mental health with this measure e.g. Sellers et al. (2019), scores of ≥17 were used to identify participants with high levels of behavioral and/or mental health problems.

Relative family poverty was assessed at age 14, and was defined as where household income was <60% of the median. Parental education was also assessed at age 14 and was defined as the main respondent's highest equivalent National UK Vocational Qualification (NVQ) level, (where 1 represents entry level and 5 represents higher degrees and postgraduate qualifications). We classified NVQ levels ≥4 as high levels of education.

Finally, we used the Short Social Provisions Scale (SPS) (Caron, 2013; Orpana et al., 2019) to measure levels of perceived social support during Waves 1 and 2 of the COVID‐19 survey. The survey used a 3‐item version of the scale, with scores ranging from 0 to 6. Longer versions of this scale with 5 and 10 items have previously been shown to have good criterion validity with positive mental health constructs, good concurrent validity with one another, as well as high internal consistency (Cronbach's alpha greater than 0.80) (Caron, 2013; Orpana et al., 2019). We classified scores ≥4 as indicating high social support.

### 2.4 Statistical analysis

We used information from the first child within each family and the main parent interviewee. We weighted the data before conducting analyzes; this weight was derived from the original sampling design weight multiplied by the web survey nonresponse weight for CW1 (Brown et al., 2020). This combined weight accounted for attrition from birth sample to CW1, as well as the intentional oversampling of specific subgroups in the original design of MCS, for example, children living in areas of greater socioeconomic deprivation, geographical areas that were more ethnically diverse, and of families living in Wales, Scotland, and Northern Ireland. Details on the efficacy of these weights can be found in Brown et al. (2020).

To explore individual changes in distress and wellbeing from Baseline to during the COVID‐19 waves of assessment (CW1/2‐aim one), we used latent change score models. The Lavaan package, version 0.6–17 (Rosseel, 2012) within R version 4.3.2 was used for these analyzes. Briefly, these models estimate latent true scores across time (here for distress and wellbeing), accounting for the measurement error associated with observed scores, and also estimate the differences between these true scores across time i.e. latent change scores (Ghisletta & McArdle, 2012; Kievit et al., 2018; Klopack & Wickrama, 2020). Model fit was considered acceptable with values lower than 3.00 for *χ* ^2^/*df*, values of 0.90 or higher for comparative fit index (CFI) and Tucker‐Lewis index (TLI), and values of 0.08 or lower for root mean square error of approximation (RMSEA) and standardized root mean square residual (SRMR) (Kline, 2023). Latent change score modeling assumes that the distance in time is equal between successive latent true scores. We therefore created two noninformative latent true scores in these models, with factor loadings fixed at 0, to model the gap between waves at ~4.5 months. These can be thought of as representing waves of assessment around August 2019 and January 2020. Missing data was handled with Full Information Maximum Likelihood estimation.

**Figure 1:**
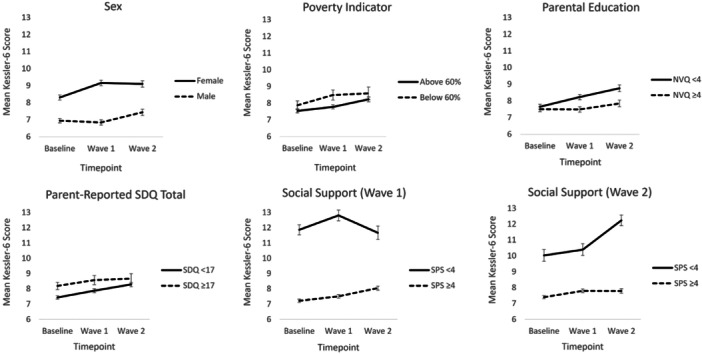
Mean observed psychological distress scores across the COVID‐19 pandemic, stratified by the vulnerability and resilience factors we tested. Error bars represent standard error of the mean.

**Figure 2:**
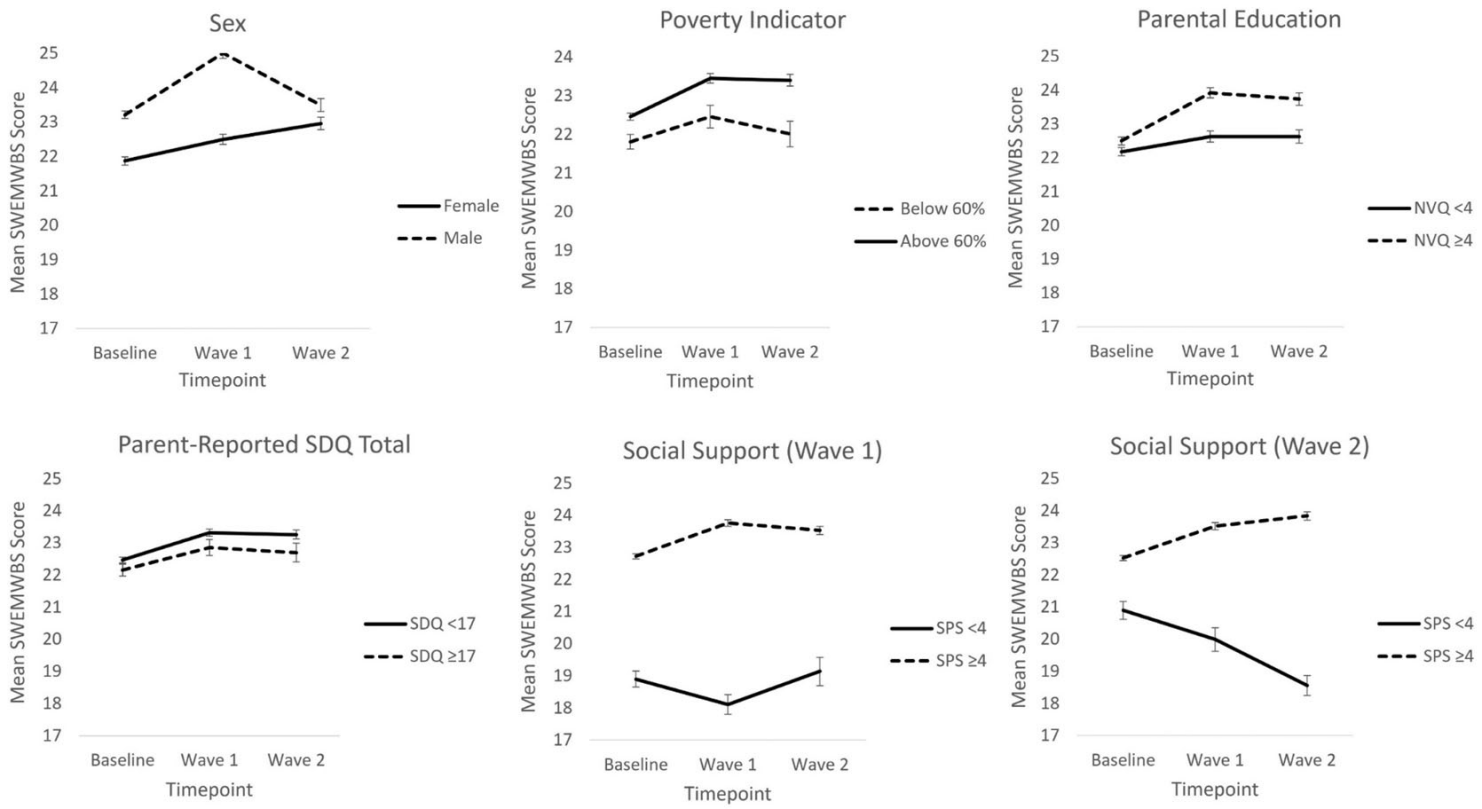
Mean observed mental wellbeing scores across the COVID‐19 pandemic, stratified by the vulnerability and resilience factors we tested. Error bars represent standard error of the mean.

For aim two of our study, we explored whether sex, relative family poverty, parental education, preexisting behavioral and/or mental health difficulties, and perceived social support were associated with (1) the intercept for distress/wellbeing (i.e., Baseline latent true score) and (2) change over time. Age at Baseline was not associated with change in either distress or wellbeing in our models and, therefore, was not included in further analyzes. Continuous measures from the SDQ (preexisting behavioral and/or mental health difficulties) and SPS (perceived social support) were used in these models, however, dichotomized versions based on cut‐off scores (≥17 for SDQ and ≥4 for SPS) have been used when presenting descriptive statistics, for illustrative purposes (Figures 1 and 2).

## 3 RESULTS

### 3.1 Change in young adult psychological distress and wellbeing across COVID‐19 (aim one)

Descriptive statistics for observed distress and wellbeing scores are provided in Table 1. For distress, weighted Ns at each wave were as follows: Baseline = 2534, CW1 = 2212, CW2 = 1480. Observed distress scores showed some mild positive skew across waves (0.49–0.65), but within acceptable bounds of ±2 (Byrne, 2013; Hair et al., 2010). For wellbeing, weighted Ns for each wave was as follows: Baseline = 2524, CW1 = 2202, CW2 = 1478. Observed wellbeing scores showed some mild positive skew at baseline (0.43), with mild negative skew at CW1 and CW2 (−0.23 to −0.24).

**Table 1:** Changes in psychological distress and mental wellbeing across the COVID‐19 pandemic in UK young adults.

	Observed score at baseline: mean (*SD*)	Observed score at COVID Wave 1: mean (*SD*)	Observed score at COVID Wave 2: mean (*SD*)	Intercept (latent true score at baseline)		LCSM: constant change		LCSM: proportional change	
				Mean, sig.	Variance, sig.	Mean, sig.	Variance, sig.	Coefficient	Sig.
Kessler‐6	7.59 (4.92)	8.00 (5.10)	8.36 (5.06)	7.61, *p* < .001	16.66, *p* < .001	1.97, *p* = .075	1.45, *p* = .214	−0.233	*p* = .098
Short WEMWBS	22.41 (4.03)	23.24 (4.89)	23.16 (4.84)	22.40, *p* < .001	8.65, *p* < .001	16.19, *p* = .022	7.85, *p* = .224	−0.697	*p* = .023

*Note*: Δdistress = 1.97−0.233 (distress_*t*−1_) and Δwellbeing = 16.19−0.697 (wellbeing_*t*−1_).

Abbreviations: LCSM, latent change score model; *SD*, standard deviation; Sig., significance; WEMWBS, Warwick‐Edinburgh Mental Wellbeing Scale.

We compared models with (1) intercept only (no change) (2) intercept plus a constant change component, (3) intercept plus a proportional change component (i.e., change is dependent on the latent true score at the previous wave), and (4) intercept plus constant change and proportional change components (i.e., a dual‐change score model). The dual‐change score models for distress and wellbeing provided the best fit to the data: distress: *χ* ^2^ = 3.07 (2), *p* = .215, RMSEA = 0.01, CFI = 0.99, TLI = 0.99, SRMR = 0.02, wellbeing: *χ* ^2^ = 1.47 (2), *p* = .479, RMSEA = 0.00, CFI = 1.00, TLI = 1.00, SRMR = 0.02. This, therefore, provides evidence of changes in both distress and wellbeing across the study period. Table 1 displays values for the intercept and change components. Participants experienced increases in both distress and wellbeing across waves (the constant change component), although the extent of this increase lessened over time (the proportional change component). For example, from Baseline to CW1, the average increase in latent true scores was estimated at 0.47 for distress and 0.80 for wellbeing. From CW1 to CW2, the average increase was estimated at 0.09 for distress and 0.02 for wellbeing.

### 3.2 Factors associated with vulnerability and resilience (aim two)

Figures 1 and 2 display mean observed scores for psychological distress and wellbeing across Baseline, CW1 and CW2, stratified by the vulnerability and resilience factors we tested. 49.67% of our sample were female, 20.86% were in relative family poverty, 47.56% had high levels of education, 19.89% scored in the abnormal range of the SDQ, and 92.03% and 92.65% had high levels of social support at CW1 and CW2, respectively.

Tables 2 and 3 shows the results from the analyzes testing which factors were associated with Baseline distress & wellbeing and change over time. For psychological distress (Table 2), female sex and a higher score on the SDQ (measure of preexisting mental health and/or behavioral difficulties) predicted higher Baseline latent true scores (i.e., initial level of distress), whereas higher perceived social support scores at CW1 and CW2 were associated with lower Baseline scores. Female sex also predicted greater increases in distress over time, whereas higher levels of parental education and perceived social support (at CW1) were associated with lesser increases. There was not strong evidence for an association with relative family poverty. Effect sizes were small‐medium, with perceived social support at CW1 showing the strongest relationship with change over time (*β* = −.43).

**Table 2:** Vulnerability and resilience factors: tests of association with baseline psychological distress scores (the intercept) and change in scores across the COVID‐19 pandemic in UK young adults.

Variable	Intercept			Constant change		
	*B* (*SE*)	Effect size *β*	*p*‐value	*B* (*SE*)	Effect size *β*	*p*‐value
Cohort member sex (0 = male, 1 = female)	1.43 (0.20)	.18	<.001	0.65 (0.28)	.23	.018
Relative poverty: 60% median poverty indicator (0 = above, 1 = below)	0.36 (0.26)	.04	.168	0.19 (0.11)	.07	.086
Parental education: highest parental NVQ Level (0 = <4, 1 = ≥4)	−0.17 (0.21)	−.02	.438	−0.27 (0.1)	− .12	.004
Preexisting mental health and/or behavioral difficulties: parent‐reported SDQ total	0.18 (0.02)	.25	<.001	0.04 (0.02)	.16	.145
Perceived social support: SPS total at CW1	−1.56 (0.09)	−.42	<.001	−0.50 (0.24)	− .43	.041
Perceived social support: SPS total at CW2	−1.03 (0.10)	−.31	<.001	−0.24 (0.17)	− .38	.150

Abbreviations: NVQ, national vocational qualification; SDQ, strengths and difficulties questionnaire; *SE*, standard error of the mean; SPS, Social Provisions Scale.

For wellbeing (Table 3) female sex, relative family poverty and higher SDQ scores predicted lower Baseline latent true scores, whereas higher scores for perceived social support at CW1 and CW2 were associated with higher Baseline wellbeing scores. Female sex and relative family poverty were associated with lesser increases in wellbeing over time, whereas higher levels of parental education and perceived social support (at CW2) were associated with greater increases. Effect sizes for associations were small‐large, with perceived social support at CW2 showing the strongest association with change over time (*β* = .56).

**Table 3:** Vulnerability and resilience factors: tests of association with baseline wellbeing scores (the intercept) and change in scores across the COVID‐19 pandemic in UK young adults.

Variable	Intercept			Constant change		
	*B* (*SE*)	Effect size *β*	*p*‐value	*B* (*SE*)	Effect size *β*	*p*‐value
Cohort member sex (0 = male, 1 = female)	−1.05 (0.17)	−.18	<.001	−0.65 (0.27)	−.13	.015
Relative poverty: 60% median poverty indicator (0 = above, 1 = below)	−0.67 (0.21)	−.09	.002	−0.84 (0.35)	−.13	.015
Parental education: highest parental NVQ Level (0 = <4, 1 = ≥4)	0.31 (0.18)	.05	.076	0.84 (0.38)	.15	.027
Preexisting mental health and/or behavioral difficulties: parent‐reported SDQ total	−0.16 (0.20)	−.32	<.001	−0.15 (2.00)	−.21	.941
Perceived social support: SPS total at CW1	1.06 (0.07)	.39	<.001	1.89 (19.53)	.51	.923
Perceived social support: SPS total at CW2	0.65 (0.09)	.27	<.001	0.72 (0.27)	.56	<.001

Abbreviations: NVQ, national vocational qualification; SDQ, strengths and difficulties questionnaire; *SE*, standard error of the mean; SPS, Social Provisions Scale.

## 4 DISCUSSION AND CONCLUSION

### 4.1 Summary and comparison with previous studies

This study explored change in psychological distress and wellbeing in a national sample of young adults in the United Kingdom, who provided a pre‐COVID assessment in 2018–2019, as well as two waves of assessment during the first year of the COVID‐19 pandemic in 2020. We also explored factors which may associate with change, with the objective of identifying vulnerable and resilient subgroups of young people. Using latent change score modeling, we observed increases in both psychological distress and wellbeing across the study period. Being female was associated with increased psychological distress and poorer wellbeing scores over time, with relative family poverty also showing an association with poorer wellbeing outcomes across this period. By contrast, young people with higher levels of parental education and higher scores for perceived social support were relatively protected against increases in distress and saw greater increases in wellbeing over time.

In line with previous research (Daly et al., 2022; Ellwardt & Präg, 2021; Kwong et al., 2021; Pierce et al., 2020; Wiedemann et al., 2022), we observed an increase in psychological distress among young adults in the United Kingdom during the first year of the COVID‐19 pandemic. However, we also found evidence for improved mental wellbeing during this period, in contrast to what has previously been found (Kauhanen et al., 2023; O'Connor et al., 2021; Wiedemann et al., 2022; Wolf & Schmitz, 2023). Previous research has demonstrated that distress and wellbeing are independent aspects of mental health; one can experience high levels of wellbeing whilst also experiencing mental health difficulties (Weich et al., 2011). Our findings provide further evidence to support this. The degree of independence between these two concepts may relate to the specific environmental challenges faced at the time of measurement (Winefield et al., 2012). The COVID‐19 pandemic and the associated restrictions are unique environmental challenges. Specifically, many young adults may have felt nervous/restless (as asked about in the K6) during this period, but may have also felt less pressure with school or work, or spent more time with family. Indeed, the quality of relationship with family and friends, as well as perceived parent supportive reactions, were found to associate with emotional adjustment during the pandemic in youth in the United States and China (Campione‐Barr et al., 2021; Shi & Wang, 2021). Indeed, in our study, perceived social support was a particularly important correlate of wellbeing during the pandemic. The average improvement in wellbeing we observed in this study suggests the impact of the COVID‐19 pandemic on young adults in the United Kingdom may not have been universally detrimental.

Our main aim was to test factors that were associated with change in mental distress and wellbeing across the COVID‐19 pandemic, to identify vulnerable and resilient subgroups of young adults. We observed that different subgroups showed heterogeneity in the impact of the COVID‐19 pandemic on their mental health and wellbeing. Females experienced poorer distress and wellbeing outcomes over the course of the study; however, the current study is unable to separate out whether this reflects normative sex differences in developmental change in emotional problems (Armitage et al., 2023; Cyranowski et al., 2000) or a differential impact related to experiences of the COVID‐19 pandemic. In fact, a previous study with an immediate pre‐COVID baseline assessment found no sex difference in increases in mental health problems associated with the pandemic in younger adolescents (Wright et al., 2021). A later study found evidence to suggest that increases in female young adolescent depressive symptoms over the COVID‐19 pandemic could be accounted for by a natural maturational rise (Wright et al., 2024). As such, our finding on sex should be interpreted with caution.

Having preexisting mental health and/or behavioral difficulties was associated with greater levels of distress and poorer wellbeing at Baseline, but did not associate with change over time. This is line with other UK studies of young adolescents e.g. Wright et al. (2021), who showed little evidence for proportional differences in rates of change of mental health disorders associated with the pandemic according to prior child emotional and behavioral problems. This does, however, contrast with the findings of other studies. For example, Wiedemann et al. (2022) found that young adults with preexisting mental health conditions such as anxiety and depression showed higher than expected psychological distress scores during the COVID‐19 pandemic. The lack of consistency of findings may reflect heterogeneity in the impacts of the pandemic on young people with different underlying vulnerabilities. Shakeshaft et al. (2023) observed that whilst anxiety symptoms increased in adults with neurodevelopmental disorders, depression symptoms decreased. During the COVID‐19 pandemic, individuals with ADHD described more time for academic work, less bullying, increased family time and more relaxation (Behrmann et al., 2021; Dvorsky et al., 2022).

We found that young people from poorer family backgrounds experienced poorer wellbeing outcomes. Low family income was highlighted as a risk factor for poor mental health outcomes in the review by Wolf and Schmitz (2023), although other studies have observed greater impacts on mental health from the pandemic in people from more advantaged backgrounds (Wright et al., 2021; Zaninotto et al., 2022). We also found that higher levels of parental education appeared protective against increased distress and poorer wellbeing, as would be expected from previous studies (Jiang et al., 2021; Ravens‐Sieberer et al., 2021; Ravens‐Sieberer et al., 2022; Schmuck et al., 2021). The degree and type of parental involvement has been shown to be strongly related to levels of maternal education (Desforges & Abouchaar, 2003).

Perceived social support also protected against increased distress and poorer wellbeing over the study period and effect sizes were largest for these associations. The importance of social support networks during periods of stress is well‐documented (Norris & Kaniasty, 1996; Ozbay et al., 2007; Taylor et al., 2004). Our findings here align with other studies in the UK and internationally, highlighting social support as an important factor for relating to mental health and wellbeing outcomes in the context of adversity; see Wolf and Schmitz (2023).

### 4.2 Limitations

The current study should be interpreted in light of several limitations. Firstly, since everybody in the UK experienced the COVID‐19 pandemic, there is no unaffected control group to compare results with. It is therefore difficult to determine if the observed increases in psychological distress (and mental wellbeing) were larger than would have been expected had our cohort not been affected by the pandemic and associated restrictions. In particular, age and exposure to the pandemic are inherently confounded, and since the Baseline and COVID‐19 pandemic assessments measured mental health in 17‐year‐olds versus 19‐year‐olds, respectively, the changes and associations observed may in fact relate to normative developmental differences in risk for mental distress, rather than reflecting experiences of the COVID‐19 pandemic specifically. It is important to note that young adulthood represents a period of increased risk for the onset of depression and anxiety (Solmi et al., 2022) and evidence suggests that once maturational change is accounted for the impacts of the COVID‐19 pandemic on young people's mental health may be more limited than suggested by simple models of change against baseline levels (Wright et al., 2024). Second, the COVID‐19 pandemic could conceivably have impacted on young people's mental health in a number of different ways, e.g. worry about exposure to the virus or for the health of others, experience of strict lockdown, closure of schools, or social restrictions leading to limited opportunities for socializing with friends. The current analyzes do not address how the COVID‐19 pandemic impacted youth distress and wellbeing. Further limitations relate to our choice of outcome measures. The K6 and SWEMWBS relate to symptoms of distress and experiences of wellbeing, rather than clinical diagnoses of depression and anxiety. In addition, the K6 does not provide separate measures of depression and anxiety, which have shown opposite trajectories across the COVID‐19 pandemic in some other studies (Hollenstein et al., 2021; Kwong et al., 2021; Shakeshaft et al., 2023). Our findings may have been different had we measured depression and anxiety separately. In addition, to our knowledge the 3‐item version of the SPS, used to measure perceived social support in MCS, has not been validated. However, a 5‐item version was recently validated, suggesting that a shortened version is sufficient to establish levels of social support (Orpana et al., 2019). The study was also limited due to the fact that only a relatively small percentage of the issued MCS sample participated in the CW1 survey (27%). Participant drop‐out/attrition is a complication within longitudinal studies, that can be problematic when there are systematic differences between those who choose to participate and those who drop out of a study. This study used a combined sampling design/nonresponse weight to more closely mirror the profile of the original population cohort, but this alone may not fully address differences between participants and non‐participants. For example, cohort members may have chosen not to participate in the COVID‐19 survey because they felt low/demotivated, because of academic pressure or because they were acting as a key worker during restrictions. These factors could be associated with poorer mental health outcomes, raising the possibility of our study underestimating the impacts of the COVID‐19 pandemic on young adult mental health. Finally, it is important to note that some COVID‐19 pandemic restrictions were beginning to lift in May 2020 (CW1); those who were unable to work from home were being encouraged to go back into work. Our findings may, therefore, have been different if the CW1 survey had been conducted in March–April 2020, when restrictions in the first UK lockdown were at their most strict.

### 4.3 Implications and future directions

Young people in relative poverty, from families with lower levels of parental education and those with low levels of social support experienced poorer outcomes in the first year of the COVID‐19 pandemic. These groups should be carefully monitored and prioritized in any interventions designed to support the recovery of poor mental health of young adults postpandemic. More generally, the study highlights the unequal impacts of adverse societal events on young people, and the likely protective role of social support in coping with such events. Future research should further explore the trajectories of psychological distress and mental wellbeing postpandemic, to better understand how to mitigate any longer‐term negative impacts on mental health and wellbeing outcomes of the generation of young people affected by the COVID‐19 pandemic.

To conclude, this study observed an increase in psychological distress and mental wellbeing in young adults in the United Kingdom, with assessments before and during the first year of the COVID‐19 pandemic. We found that being female and in relative poverty were associated with increased distress and/or poorer wellbeing, whilst higher levels of both parent education and perceived social support protected against poorer outcomes. In particular, young adults with low levels of perceived social support at the time of the pandemic should be a priority group for policies and interventions designed to support postpandemic mental health moving forward.

## CONFLICT OF INTEREST STATEMENT

The authors declare no conflicts of interest.

## ETHICS STATEMENT

The Millenium Cohort Study received ethical approval from the London Multi‐Centre Research Ethics Committee.

## Data Availability

The data used are publicly accessible via the UK Data Service (study numbers: 4683, 8156, 8658, 8682).

## References

[jad12400-bib-0001] Alt, P. , Reim, J. , & Walper, S. (2021). Fall from grace: Increased loneliness and depressiveness among extraverted youth during the German COVID‐19 lockdown. Journal of Research on Adolescence, 31(3), 678–691. 34448311 10.1111/jora.12648 PMC8646507

[jad12400-bib-0002] Armitage, J. M. , Kwong, A. S. F. , Tseliou, F. , Sellers, R. , Blakey, R. , Anthony, R. , Rice, F. , Thapar, A. , & Collishaw, S. (2023). Cross‐cohort change in parent‐reported emotional problem trajectories across childhood and adolescence in the UK. The Lancet Psychiatry, 10(7), 509–517. 37244272 10.1016/S2215-0366(23)00175-X

[jad12400-bib-0003] Behrmann, J. T. , Blaabjerg, J. , Jordansen, J. , & Jensen de López, K. M. (2021). Systematic review: Investigating the impact of COVID‐19 on mental health outcomes of individuals with ADHD. Journal of attention disorders, 26(7), 959–975. 10.1177/10870547211050945 34654341

[jad12400-bib-0004] Bélanger, R. E. , Patte, K. A. , Leatherdale, S. T. , Gansaonré, R. J. , & Haddad, S. (2021). An impact analysis of the early months of the COVID‐19 pandemic on mental health in a prospective cohort of Canadian adolescents. Journal of Adolescent Health, 69(6), 917–924. 10.1016/j.jadohealth.2021.07.039 PMC8457891 34565667

[jad12400-bib-0005] Bernasco, E. L. , Nelemans, S. A. , van der Graaff, J. , & Branje, S. (2021). Friend support and internalizing symptoms in early adolescence during COVID‐19. Journal of Research on Adolescence, 31(3), 692–702. 34448295 10.1111/jora.12662 PMC8457148

[jad12400-bib-0006] Brown, M. , Goodman, A. , Peters, A. , Ploubidis, G. , Aida, S. , Silverwood, R. , & Smith, K. (2020). COVID‐19 survey in five national longitudinal studies: Waves 1 and 2: User guide (version 2).

[jad12400-bib-0007] Byrne, B. M. (2013). Structural equation modeling with Mplus: Basic concepts, applications, and programming. routledge.

[jad12400-bib-0008] Campione‐Barr, N. , Rote, W. , Killoren, S. E. , & Rose, A. J. (2021). Adolescent adjustment during COVID‐19: The role of close relationships and COVID‐19‐related stress. Journal of Research on Adolescence, 31(3), 608–622. 34448310 10.1111/jora.12647 PMC8646630

[jad12400-bib-0009] Caron, J. (2013). A validation of the Social Provisions Scale: The SPS‐10 items [article in French]. Sante Ment Que, 38(1), 297–318. 10.7202/1019198ar PMC5031489 24337002

[jad12400-bib-0010] Chen, Y. , Osika, W. , Henriksson, G. , Dahlstrand, J. , & Friberg, P. (2021). Impact of COVID‐19 pandemic on mental health and health behaviors in Swedish adolescents. Scandinavian Journal of Public Health, 50(1), 26–32. 10.1177/14034948211021724 34100665 PMC8808000

[jad12400-bib-0011] Collishaw, S. , & Sellers, R. (2020). Trends in child and adolescent mental health prevalence, outcomes, and inequalities. Mental Health and Illness of Children and Adolescents, 63–73. 10.1007/978-981-10-2348-4_9

[jad12400-bib-0012] Connelly, R. , & Platt, L. (2014). Cohort profile: UK millennium cohort study (MCS). International Journal of Epidemiology, 43(6), 1719–1725. 24550246 10.1093/ije/dyu001

[jad12400-bib-0013] Cyranowski, J. M. , Frank, E. , Young, E. , & Shear, M. K. (2000). Adolescent onset of the gender difference in lifetime rates of major depression: A theoretical model. Archives of General Psychiatry, 57(1), 21–27. 10632229 10.1001/archpsyc.57.1.21

[jad12400-bib-0014] Daly, M. , Sutin, A. R. , & Robinson, E. (2022). Longitudinal changes in mental health and the COVID‐19 pandemic: Evidence from the UK household longitudinal study. Psychological Medicine, 52(13), 2549–2558. 33183370 10.1017/S0033291720004432 PMC7737138

[jad12400-bib-0015] Deng, W. , Gadassi Polack, R. , Creighton, M. , Kober, H. , & Joormann, J. (2021). Predicting negative and positive affect during COVID‐19: A daily diary study in youths. Journal of Research on Adolescence, 31(3), 500–516. 34448307 10.1111/jora.12646 PMC8646745

[jad12400-bib-0016] Desforges, C. , & Abouchaar, A. (2003). The impact of parental involvement, parental support and family education on pupil achievement and adjustment: A literature review (433). DfES.

[jad12400-bib-0017] Dvorsky, M. R. , Breaux, R. , Cusick, C. N. , Fredrick, J. W. , Green, C. , Steinberg, A. , Langberg, J. M. , Sciberras, E. , & Becker, S. P. (2022). Coping with COVID‐19: Longitudinal impact of the pandemic on adjustment and links with coping for adolescents with and without ADHD. Research on Child and Adolescent Psychopathology, 50(5), 605–619. 34618271 10.1007/s10802-021-00857-2 PMC8496139

[jad12400-bib-0018] Ellwardt, L. , & Präg, P. (2021). Heterogeneous mental health development during the COVID‐19 pandemic in the United Kingdom. Scientific Reports, 11(1), 15958. 34354201 10.1038/s41598-021-95490-w PMC8342469

[jad12400-bib-0019] Ghisletta, P. , & McArdle, J. J. (2012). Teacher's corner: Latent curve models and latent change score models estimated in R. Structural Equation Modeling: A Multidisciplinary Journal, 19(4), 651–682. 25505366 10.1080/10705511.2012.713275 PMC4259494

[jad12400-bib-0020] Gibb, S. J. , Fergusson, D. M. , & Horwood, L. J. (2010). Burden of psychiatric disorder in young adulthood and life outcomes at age 30. British Journal of Psychiatry, 197(2), 122–127. 10.1192/bjp.bp.109.076570 20679264

[jad12400-bib-0021] Di Giunta, L. , Lunetti, C. , Fiasconaro, I. , Gliozzo, G. , Salvo, G. , Ottaviani, C. , Aringolo, K. , Comitale, C. , Riccioni, C. , & D'Angeli, G. (2021). COVID‐19 impact on parental emotion socialization and youth socioemotional adjustment in Italy. Journal of Research on Adolescence, 31(3), 657–677. 34448309 10.1111/jora.12669 PMC8646882

[jad12400-bib-0022] Goodman, R. (2001). Psychometric properties of the strengths and difficulties questionnaire. Journal of the American Academy of Child and Adolescent Psychiatry, 40(11), 1337–1345. 11699809 10.1097/00004583-200111000-00015

[jad12400-bib-0023] Hair, J. F. , Anderson, R. E. , Babin, B. J. , & Black, W. C. (2010). In: Multivariate data analysis: A global perspective (7). Pearson.

[jad12400-bib-0024] Hollenstein, T. , Colasante, T. , & Lougheed, J. P. (2021). Adolescent and maternal anxiety symptoms decreased but depressive symptoms increased before to during COVID‐19 lockdown. Journal of Research on Adolescence, 31(3), 517–530. 34448298 10.1111/jora.12663 PMC8646576

[jad12400-bib-0026] Jiang, H. , Yu, W. , Lin, D. , & Macnamara, B. N. (2021). Resilience of adolescents, though weakened during pandemic‐related lockdown, serves as a protection against depression and sleep problems. Psychology, Health & Medicine, 27(9), 1977–1988. 10.1080/13548506.2021.1990367 34663152

[jad12400-bib-0027] Johansson, F. , Côté, P. , Hogg‐Johnson, S. , & Skillgate, E. (2021). Depression, anxiety and stress among Swedish university students during the second and third waves of COVID‐19: A cohort study. Scandinavian Journal of Public Health, 49(7), 750–754. 34304621 10.1177/14034948211031402 PMC8521365

[jad12400-bib-0028] Johnson, D. , Dupuis, G. , Piche, J. , Clayborne, Z. , & Colman, I. (2018). Adult mental health outcomes of adolescent depression: A systematic review. Depression and Anxiety, 35(8), 700–716. 29878410 10.1002/da.22777

[jad12400-bib-0029] Kauhanen, L. , Wan Mohd Yunus Mohd Yunus, W. , Lempinen, L. , Peltonen, K. , Gyllenberg, D. , Mishina, K. , Gilbert, S. , Bastola, K. , Brown, J. , & Sourander, A. (2023). A systematic review of the mental health changes of children and young people before and during the COVID‐19 pandemic. European Child & Adolescent Psychiatry, 32(6), 995–1013. 35962147 10.1007/s00787-022-02060-0 PMC9373888

[jad12400-bib-0030] Kessler, R. C. , Andrews, G. , Colpe, L. J. , Hiripi, E. , Mroczek, D. K. , Normand, S. L. T. , Walters, E. E. , & Zaslavsky, A. M. (2002). Short screening scales to monitor population prevalences and trends in non‐specific psychological distress. Psychological Medicine, 32(6), 959–976. 12214795 10.1017/s0033291702006074

[jad12400-bib-0031] Kessler, R. C. , Barker, P. R. , Colpe, L. J. , Epstein, J. F. , Gfroerer, J. C. , Hiripi, E. , Howes, M. J. , Normand, S. L. T. , Manderscheid, R. W. , Walters, E. E. , & Zaslavsky, A. M. (2003). Screening for serious mental illness in the general population. Archives of General Psychiatry, 60(2), 184–189. 12578436 10.1001/archpsyc.60.2.184

[jad12400-bib-0032] Kessler, R. C. , Berglund, P. , Demler, O. , Jin, R. , Merikangas, K. R. , & Walters, E. E. (2005). Lifetime prevalence and age‐of‐onset distributions of DSM‐IV disorders in the national comorbidity survey replication. Archives of General Psychiatry, 62(6), 593–602. 15939837 10.1001/archpsyc.62.6.593

[jad12400-bib-0033] Kievit, R. A. , Brandmaier, A. M. , Ziegler, G. , van Harmelen, A.‐L. , de Mooij, S. M. M. , Moutoussis, M. , Goodyer, I. M. , Bullmore, E. , Jones, P. B. , Fonagy, P. , Lindenberger, U. , & Dolan, R. J. (2018). Developmental cognitive neuroscience using latent change score models: A tutorial and applications. Developmental Cognitive Neuroscience, 33, 99–117. 29325701 10.1016/j.dcn.2017.11.007 PMC6614039

[jad12400-bib-0034] Kline, R. B. (2023). Principles and practice of structural equation modeling. Guilford publications.

[jad12400-bib-0035] Klopack, E. T. , & Wickrama, K. K. A. S. (2020). Modeling latent change score analysis and extensions in Mplus: A practical guide for researchers. Structural Equation Modeling: A Multidisciplinary Journal, 27(1), 97–110. 33013155 10.1080/10705511.2018.1562929 PMC7531193

[jad12400-bib-0036] Kwong, A. S. F. , Pearson, R. M. , Adams, M. J. , Northstone, K. , Tilling, K. , Smith, D. , Fawns‐Ritchie, C. , Bould, H. , Warne, N. , Zammit, S. , Gunnell, D. J. , Moran, P. A. , Micali, N. , Reichenberg, A. , Hickman, M. , Rai, D. , Haworth, S. , Campbell, A. , Altschul, D. , … Timpson, N. J. (2021). Mental health before and during the COVID‐19 pandemic in two longitudinal UK population cohorts. The British Journal of Psychiatry, 218(6), 334–343. 33228822 10.1192/bjp.2020.242 PMC7844173

[jad12400-bib-0037] Lu, P. , Yang, L. , Wang, C. , Xia, G. , Xiang, H. , Chen, G. , Jiang, N. , Ye, T. , Pang, Y. , Sun, H. , Yan, L. , Su, Z. , Heyworth, J. , Huxley, R. , Fisher, J. , Li, S. , & Guo, Y. (2021). Mental health of new undergraduate students before and after COVID‐19 in China. Scientific Reports, 11(1), 18783. 10.1038/s41598-021-98140-3 34552105 PMC8458482

[jad12400-bib-0038] Luijten, M. A. J. , van Muilekom, M. M. , Teela, L. , Polderman, T. J. C. , Terwee, C. B. , Zijlmans, J. , Klaufus, L. , Popma, A. , Oostrom, K. J. , van Oers, H. A. , & Haverman, L. (2021). The impact of lockdown during the COVID‐19 pandemic on mental and social health of children and adolescents. Quality of Life Research, 30(10), 2795–2804. 33991278 10.1007/s11136-021-02861-x PMC8122188

[jad12400-bib-0039] Magson, N. R. , Freeman, J. Y. A. , Rapee, R. M. , Richardson, C. E. , Oar, E. L. , & Fardouly, J. (2021). Risk and protective factors for prospective changes in adolescent mental health during the COVID‐19 pandemic. Journal of Youth and Adolescence, 50(1), 44–57. 10.1007/s10964-020-01332-9 33108542 PMC7590912

[jad12400-bib-0040] Norris, F. H. , & Kaniasty, K. (1996). Received and perceived social support in times of stress: A test of the social support deterioration deterrence model. Journal of Personality and Social Psychology, 71(3), 498–511. 10.1037/0022-3514.71.3.498 8831159

[jad12400-bib-0041] O'Connor, R. C. , Wetherall, K. , Cleare, S. , McClelland, H. , Melson, A. J. , Niedzwiedz, C. L. , O'Carroll, R. E. , O'Connor, D. B. , Platt, S. , Scowcroft, E. , Watson, B. , Zortea, T. , Ferguson, E. , & Robb, K. A. (2021). Mental health and well‐being during the COVID‐19 pandemic: Longitudinal analyses of adults in the UK COVID‐19 mental health & wellbeing study. The British Journal of Psychiatry, 218(6), 326–333. 33081860 10.1192/bjp.2020.212 PMC7684009

[jad12400-bib-0042] Orpana, H. M. , Lang, J. J. , & Yurkowski, K. (2019). Validation of a brief version of the Social Provisions Scale using Canadian national survey data. Health Promotion and Chronic Disease Prevention in Canada, 39(12), 323–332. 10.24095/hpcdp.39.12.02 31825785 PMC6938275

[jad12400-bib-0043] Ozbay, F. , Johnson, D. C. , Dimoulas, E. , Morgan, C. A. , Charney, D. , & Southwick, S. (2007). Social support and resilience to stress: From neurobiology to clinical practice. Psychiatry (Edgmont (Pa.: Township)), 4(5), 35–40. Retrieved from https://pubmed.ncbi.nlm.nih.gov/20806028 PMC2921311 20806028

[jad12400-bib-0044] Pierce, M. , Hope, H. , Ford, T. , Hatch, S. , Hotopf, M. , John, A. , Kontopantelis, E. , Webb, R. , Wessely, S. , McManus, S. , & Abel, K. M. (2020). Mental health before and during the COVID‐19 pandemic: A longitudinal probability sample survey of the UK population. The Lancet Psychiatry, 7(10), 883–892. 10.1016/S2215-0366(20)30308-4 32707037 PMC7373389

[jad12400-bib-0045] Ravens‐Sieberer, U. , Kaman, A. , Erhart, M. , Devine, J. , Schlack, R. , & Otto, C. (2022). Impact of the COVID‐19 pandemic on quality of life and mental health in children and adolescents in Germany. European Child & Adolescent Psychiatry, 31(6), 879–889. 33492480 10.1007/s00787-021-01726-5 PMC7829493

[jad12400-bib-0046] Ravens‐Sieberer, U. , Kaman, A. , Erhart, M. , Otto, C. , Devine, J. , Löffler, C. , Hurrelmann, K. , Bullinger, M. , Barkmann, C. , Siegel, N. A. , Simon, A. M. , Wieler, L. H. , Schlack, R. , & Hölling, H. (2021). Quality of life and mental health in children and adolescents during the first year of the COVID‐19 pandemic: Results of a two‐wave nationwide population‐based study. European Child & Adolescent Psychiatry, 32(4), 575–588. 10.1007/s00787-021-01889-1 34636964 PMC8506100

[jad12400-bib-0047] Romm, K. F. , Park, Y. W. , Hughes, J. L. , & Gentzler, A. L. (2021). Risk and protective factors for changes in adolescent psychosocial adjustment during COVID‐19. Journal of Research on Adolescence, 31(3), 546–559. 34448304 10.1111/jora.12667 PMC8646485

[jad12400-bib-0048] Rosa, L. , Godwin, H. J. , Cortese, S. , & Brandt, V. (2022). Predictors of longer‐term depression trajectories during the COVID‐19 pandemic: A longitudinal study in four UK cohorts. Evidence Based Mental Health, 25(4), e3. 35902216 10.1136/ebmental-2022-300461 PMC10231611

[jad12400-bib-0049] Rosseel, Y. (2012). lavaan: An R package for structural equation modeling. Journal of Statistical Software, 48, 1–36.

[jad12400-bib-0050] Sabato, H. , Abraham, Y. , & Kogut, T. (2021). Too lonely to help: Early adolescents' social connections and willingness to help during COVID‐19 lockdown. Journal of Research on Adolescence, 31(3), 764–779. 34448302 10.1111/jora.12655 PMC8646666

[jad12400-bib-1000] Sadler, K. , Vizard, T. , Ford, T. , Goodman, A. , Goodman, R. , & McManus, S. (2018). Mental health of children and young people in England, 2017: Trends and characteristics.

[jad12400-bib-0052] Schmuck, J. , Hiebel, N. , Rabe, M. , Schneider, J. , Erim, Y. , Morawa, E. , Jerg‐Bretzke, L. , Beschoner, P. , Albus, C. , Hannemann, J. , Weidner, K. , Steudte‐Schmiedgen, S. , Radbruch, L. , Brunsch, H. , & Geiser, F. (2021). Sense of coherence, social support and religiosity as resources for medical personnel during the COVID‐19 pandemic: A web‐based survey among 4324 health care workers within the German Network University Medicine. PLoS One, 16(7), e0255211‐e0255211. 10.1371/journal.pone.0255211 34310616 PMC8312980

[jad12400-bib-0053] Sellers, R. , Warne, N. , Pickles, A. , Maughan, B. , Thapar, A. , & Collishaw, S. (2019). Cross‐cohort change in adolescent outcomes for children with mental health problems. Journal of Child Psychology and Psychiatry, 60(7), 813–821. 30989670 10.1111/jcpp.13029 PMC6617990

[jad12400-bib-0054] Shakeshaft, A. , Blakey, R. , Kwong, A. S. F. , Riglin, L. , Davey Smith, G. , Stergiakouli, E. , Tilling, K. , & Thapar, A. (2023). Mental‐health before and during the COVID‐19 pandemic in adults with neurodevelopmental disorders. Journal of Psychiatric Research, 159, 230–239. 36753897 10.1016/j.jpsychires.2023.01.029 PMC9885110

[jad12400-bib-0055] Shi, Z. , & Wang, Q. (2021). Chinese adolescents' coping with COVID‐19: Relationships with emotional maladjustment and parental reactions to negative emotions. Journal of Research on Adolescence, 31(3), 645–656. 34448290 10.1111/jora.12649 PMC8646946

[jad12400-bib-0056] Shoshani, A. , & Kor, A. (2021). The mental health effects of the COVID‐19 pandemic on children and adolescents: Risk and protective factors. Psychological Trauma: Theory, Research, Practice, and Policy, 14(8), 1365–1373. 34928689 10.1037/tra0001188

[jad12400-bib-0057] Sigfusdottir, I. D. , Asgeirsdottir, B. B. , Sigurdsson, J. F. , & Gudjonsson, G. H. (2008). Trends in depressive symptoms, anxiety symptoms and visits to healthcare specialists: A national study among Icelandic adolescents. Scandinavian Journal of Public Health, 36(4), 361–368. 18539690 10.1177/1403494807088457

[jad12400-bib-0058] Solmi, M. , Radua, J. , Olivola, M. , Croce, E. , Soardo, L. , Salazar de Pablo, G. , Il Shin, J. , Kirkbride, J. B. , Jones, P. , Kim, J. H. , Kim, J. Y. , Carvalho, A. F. , Seeman, M. V. , Correll, C. U. , & Fusar‐Poli, P. (2022). Age at onset of mental disorders worldwide: Large‐scale meta‐analysis of 192 epidemiological studies. Molecular Psychiatry, 27(1), 281–295. 34079068 10.1038/s41380-021-01161-7 PMC8960395

[jad12400-bib-0059] Stewart‐Brown, S. , Tennant, A. , Tennant, R. , Platt, S. , Parkinson, J. , & Weich, S. (2009). Internal construct validity of the Warwick‐Edinburgh mental well‐being scale (WEMWBS): A Rasch analysis using data from the Scottish health education population survey. Health and Quality of Life Outcomes, 7(1), 15. 19228398 10.1186/1477-7525-7-15 PMC2669062

[jad12400-bib-0060] Stroud, I. , & Gutman, L. M. (2021). Longitudinal changes in the mental health of UK young male and female adults during the COVID‐19 pandemic. Psychiatry Research, 303, 114074. 10.1016/j.psychres.2021.114074 34271372 PMC8520320

[jad12400-bib-0061] Taylor, S. E. , Sherman, D. K. , Kim, H. S. , Jarcho, J. , Takagi, K. , & Dunagan, M. S. (2004). Culture and social support: Who seeks it and why. Journal of Personality and Social Psychology, 87(3), 354–362. 10.1037/0022-3514.87.3.354 15382985

[jad12400-bib-0062] Thapar, A. , Collishaw, S. , Pine, D. S. , & Thapar, A. K. (2012). Depression in adolescence. The Lancet, 379(9820), 1056–1067. 10.1016/S0140-6736(11)60871-4 PMC3488279 22305766

[jad12400-bib-0063] Umucu, E. , Fortuna, K. , Jung, H. , Bialunska, A. , Lee, B. , Mangadu, T. , Storm, M. , Ergun, G. , Mozer, D. A. , & Brooks, J. (2021). A national study to assess validity and psychometrics of the Short Kessler Psychological Distress Scale (K6). Rehabilitation Counseling Bulletin, 65(2), 140–149. 10.1177/00343552211043261 39301105 PMC11412066

[jad12400-bib-0064] Vaingankar, J. A. , Abdin, E. , Chong, S. A. , Sambasivam, R. , Seow, E. , Jeyagurunathan, A. , Picco, L. , Stewart‐Brown, S. , & Subramaniam, M. (2017). Psychometric properties of the short Warwick Edinburgh mental well‐being scale (SWEMWBS) in service users with schizophrenia, depression and anxiety spectrum disorders. Health and Quality of Life Outcomes, 15, 153. 28764770 10.1186/s12955-017-0728-3 PMC5539899

[jad12400-bib-2000] Viner, R. , Russell, S. , Saulle, R. , Croker, H. , Stansfield, C. , Packer, J. , Nicholls, D. , Goddings, A. L. , Bonell, C. , Hudson, L. , Hope, S. , Ward, J. , Schwalbe, N. , Morgan, A. , & Minozzi, S. (2022). School Closures During Social Lockdown and Mental Health, Health Behaviors, and Well‐being Among Children and Adolescents During the First COVID‐19 Wave: A Systematic Review. JAMA pediatrics, 176(4), 400–409. 10.1001/jamapediatrics.2021.5840 35040870

[jad12400-bib-0065] Vira, E. G. , & Skoog, T. (2021). Swedish middle school students' psychosocial well‐being during the COVID‐19 pandemic: A longitudinal study. SSM ‐ Population Health, 16, 100942. 10.1016/j.ssmph.2021.100942 34664029 PMC8516135

[jad12400-bib-0066] Weich, S. , Brugha, T. , King, M. , McManus, S. , Bebbington, P. , Jenkins, R. , Cooper, C. , McBride, O. , & Stewart‐Brown, S. (2011). Mental well‐being and mental illness: findings from the adult psychiatric morbidity survey for England 2007. British Journal of Psychiatry, 199(1), 23–28. 10.1192/bjp.bp.111.091496 21719878

[jad12400-bib-0067] Wiedemann, A. , Stochl, J. , Neufeld, S. A. S. , Fritz, J. , Bhatti, J. , Hook, R. W. , Consortium, N. , Bullmore, E. , Dolan, R. , Goodyer, I. , Fonagy, P. , Jones, P. , Moutoussis, M. , Hauser, T. , Neufeld, S. , Romero‐Garcia, R. , Clair, M. S. , Vértes, P. , Whitaker, K. , Inkster, B. , … Jones, P. B. (2022). The impact of the initial COVID‐19 outbreak on young adults' mental health: A longitudinal study of risk and resilience factors. Scientific Reports, 12(1), 16659. 10.1038/s41598-022-21053-2 36198725 PMC9533974

[jad12400-bib-0068] Winefield, H. R. , Gill, T. K. , Taylor, A. W. , & Pilkington, R. M. (2012). Psychological well‐being and psychological distress: Is it necessary to measure both. Psychology of Well‐Being: Theory, Research and Practice, 2(1), 3. 10.1186/2211-1522-2-3

[jad12400-bib-0069] Wolf, K. , & Schmitz, J. (2023). Scoping review: Longitudinal effects of the COVID‐19 pandemic on child and adolescent mental health. European Child & Adolescent Psychiatry, 33(5), 1–56. 10.1007/s00787-023-02206-8 PMC10119016 37081139

[jad12400-bib-0070] Wright, N. , Hill, J. , Sharp, H. , & Pickles, A. (2021). Interplay between long‐term vulnerability and new risk: Young adolescent and maternal mental health immediately before and during the COVID‐19 pandemic. JCPP Advances, 1(1), e12008. 34485987 10.1111/jcv2.12008 PMC8206735

[jad12400-bib-0071] Wright, N. , Hill, J. , Sharp, H. , Refberg‐Brown, M. , Crook, D. , Kehl, S. , & Pickles, A. (2024). COVID‐19 pandemic impact on adolescent mental health: A reassessment accounting for development. European Child & Adolescent Psychiatry, 33(8), 2615–2627. 38170282 10.1007/s00787-023-02337-y PMC11272811

[jad12400-bib-0072] Zaninotto, P. , Iob, E. , Demakakos, P. , & Steptoe, A. (2022). Immediate and longer‐term changes in the mental health and well‐being of older adults in England during the COVID‐19 pandemic. JAMA Psychiatry, 79(2), 151–159. 34935862 10.1001/jamapsychiatry.2021.3749 PMC8696687

